# A live attenuated vaccine prevents replication and transmission of H7N9 virus in mammals

**DOI:** 10.1038/srep11233

**Published:** 2015-06-10

**Authors:** Huihui Kong, Qianyi Zhang, Chunyang Gu, Jianzhong Shi, Guohua Deng, Shujie Ma, Jinxiong Liu, Pucheng Chen, Yuntao Guan, Yongping Jiang, Hualan Chen

**Affiliations:** 1 State Key Laboratory of Veterinary Biotechnology, Harbin Veterinary Research Institute, Chinese Academy of Agricultural Sciences, Harbin 150001, People’s Republic of China; 2 Department of Inspection Technology Research & National Classical Swine Fever Reference Laboratory, China Institute of Veterinary Drug Control, Beijing 100081, People’s Republic of China

## Abstract

The continued spread of the newly emerged H7N9 viruses among poultry in China, together with the emergence of drug-resistant variants and the possibility of human-to-human transmission, has spurred attempts to develop an effective vaccine. An MF59-adjuvant H7N9 inactivated vaccine is reported to be well-tolerated and immunogenic in humans; however a study in ferrets indicated that while a single dose of the inactivated H7N9 vaccine reduced disease severity, it did not prevent virus replication and transmission. In this study, we used reverse genetics to produce a cold-adapted, live attenuated H7N9 vaccine (H7N9/AA*ca*) that contains wild-type HA and NA genes from AH/1, and the backbone of the cold-adapted influenza H2N2 A/Ann Arbor/6/60 virus (AA*ca*). H7N9/AA*ca* was attenuated in mice and ferrets, and induced robust neutralizing antibody responses in rhesus mice, ferrets, and guinea pigs immunized once or twice intranasally. The animals immunized twice were completely protected from H7N9 virus challenge. Importantly, the animals vaccinated once were fully protected from transmission when exposed to or in contact with the H7N9 virus-inoculated animals. These results demonstrate that a cold-adapted H7N9 vaccine can prevent H7N9 virus transmission; they provide a compelling argument for further testing of this vaccine in human trials.

Influenza viruses are divided into subtypes on the basis of the antigenicity of their surface glycoproteins, hemagglutinin (HA) and neuraminidase (NA); currently 16 HA and 9 NA subtypes of type A influenza virus have been isolated from birds. Except for the highly pathogenic H5 and H7 subtype viruses, which can cause severe disease outbreaks in poultry, influenza viruses are non or low pathogenic in poultry and therefore are frequently neglected in animal disease control; however, both the highly pathogenic and the low pathogenic avian influenza viruses pose risks for human public health^1^,^2^,^3^,^4^,^5^,^6^.

The H7N9 influenza viruses emerged in Eastern China, silently replicating in chickens without causing disease; however, when they replicate in humans, these viruses can acquire more mutations, including the changes of 627 K and 701N in PB2, and become more virulent in the human population^7^,^8^,^9^,^10^. A recent study reported that a novel H7N2 virus bearing seven gene segments of the H7N9 virus and the NA gene of an H9N2 virus replicated more efficiently in mice than did the H7N9 avian influenza virus^11^. Despite substantial efforts to control the infection in poultry, H7N9 viruses have continued to evolve and spread, producing human infections in 16 provinces of inland China, Taiwan, and Hong Kong, with 238 of the 625 confirmed cases proving fatal as of March 1, 2015 (http://www.who.int/influenza/human_animal_interface/influenza_h7n9/ en/index.html). Studies indicate that the H7N9 viruses can bind human-type receptors and transmit in ferrets^12^,^13^,^14^,^15^,^16^, and therefore they pose a huge risk for a new human influenza pandemic. In addition, the emergence of H7N9 viruses resistant to adamantanes and oseltamivir^17^ has raised serious concerns over the ability of current antiviral agents to prevent global influenza outbreaks. Thus, the development of an effective vaccine has assumed the highest priority in H7N9 influenza pandemic preparedness.

An H7N9 monovalent vaccine with the MF59-adjuvant was shown to be tolerable and immunogenic in adults; two doses of the vaccine induced potentially protective immune responses in most of the subjects with no pre-existing immunity to the H7N9 virus^18^. An animal study, however, demonstrated that one dose of the H7N9 inactivated vaccine could not prevent the replication and transmission of the wild-type H7N9 virus in ferrets^19^. Live attenuated vaccines can induce humoral, cellular, and mucosal immunity and generally induce broadly cross-reactive protection^20^,^21^. They are also easier to produce than inactivated vaccines, and they do not require adjuvants. Accordingly, these vaccines may be a useful tool in an emerging pandemic. The cold-adapted influenza virus A/Ann Arbor/6/60 (H2N2) (AA*ca*) has been developed and used as an internal gene donor for live attenuated seasonal influenza vaccine seed viruses in the United States for many years. Live attenuated vaccines with HA and NA gene segments from candidate viruses of several different subtypes of influenza viruses with pandemic potential and their remaining six gene segments from AA*ca* have been developed and evaluated in mice, ferrets, and nonhuman primates^22^,^23^,^24^,^25^,^26^,^27^,^28^,^29^. Chen *et al.* reported that an H7N9 AA*ca* reassortant virus bearing two amino acid changes in HA could prevent H7N9 virus replication in ferrets^30^.

In this study, we generated a live attenuated H7N9 vaccine seed virus bearing the HA and NA gene segments from A/Anhui/1/2013 (H7N9) (AH/1) and its remaining six gene segments from AA*ca,* by using plasmid-based reverse genetics^31^,^32^,^33^. We evaluated the immunogenicity and protective capacity of the live H7N9/AA*ca* vaccine in mice, ferrets, and guinea pigs. We also investigated whether a single dose of the vaccine could prevent H7N9 virus transmission in mammals.

## Results

### Characterization of the reassortant H7N9/AA*ca*

The reassortant virus H7N9/AA*ca* with its HA and NA genes derived from AH/1 and its remaining gene segments derived from AA*ca* was generated by using reverse genetics^31^,^32^,^33^. Sequencing analysis of the surface and internal genes confirmed the expected genotype of the reassortant.

**Figure 1:**
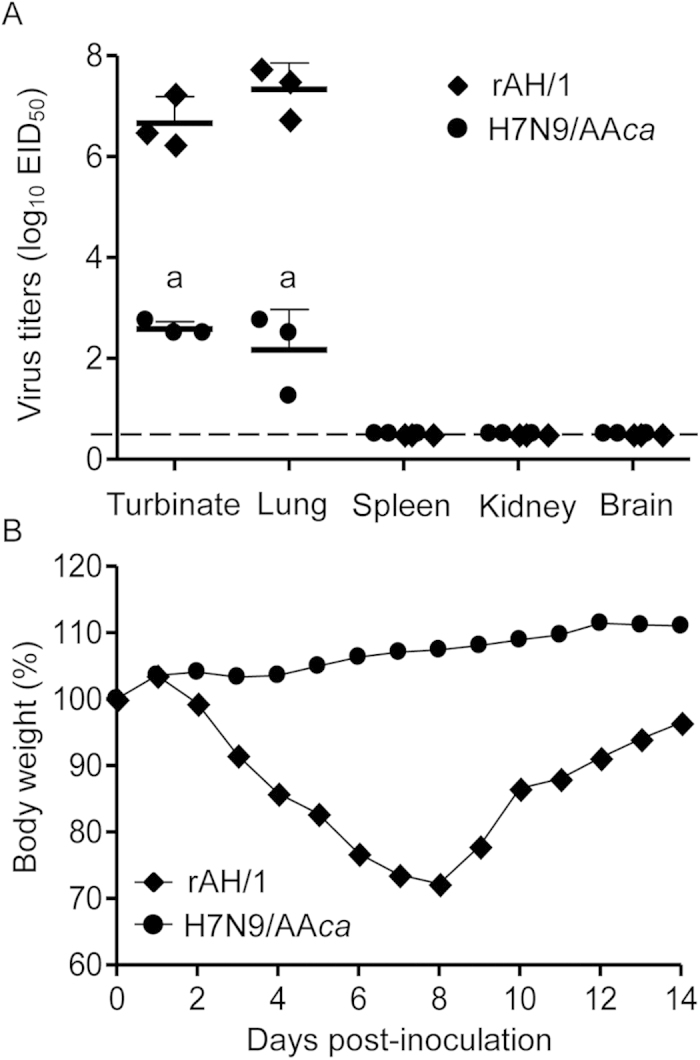
Attenuation of H7N9/AAca in mice. Groups of eight mice were intranasally inoculated with 10^6^ EID_50_ of H7N9/AA*ca* or rAH/1. Three mice in each group were killed on day 3 p.i. and nasal turbinates, lungs, spleens, kidneys, and brains were collected for virus titration in eggs (**A**). The remaining five mice were observed for weight changes over 14 days (**B**). The dashed line indicates the limit of detection. a, *P* < 0.001 compared with the corresponding value for rAH/1-inoculated animals.

Our previous study indicated that the AH/1 virus replicated efficiently in the respiratory system of mice and in multiple organs of ferrets^12^. To investigate whether the reassortant virus H7N9/AA*ca* bearing the internal genes of the AA*ca* virus is attenuated in mammals, we compared the replication of H7N9/AA*ca* and rAH/1 in both mice and ferrets. rAH/1 virus replicated in mice with mean titers of 6.7 log_10_ EID_50_ and 7.3 log_10_ EID_50_ in the nasal turbinates and lungs, respectively (Fig. 1A). In the H7N9/AA*ca-*inoculated mice, the mean viral titers were 2.6 log_10_ EID_50_ and 2.2 log_10_ EID_50_ in the turbinates and lungs, respectively, which were significantly lower than those in the rAH/1-inoculated mice. The rAH/1-inoculated mice lost up to 28% of their body weight during the two-week observation period; in contrast, the mice inoculated with the H7N9/AA*ca* virus gained weight during the observation period (Fig. 1B).

**Figure 2:**
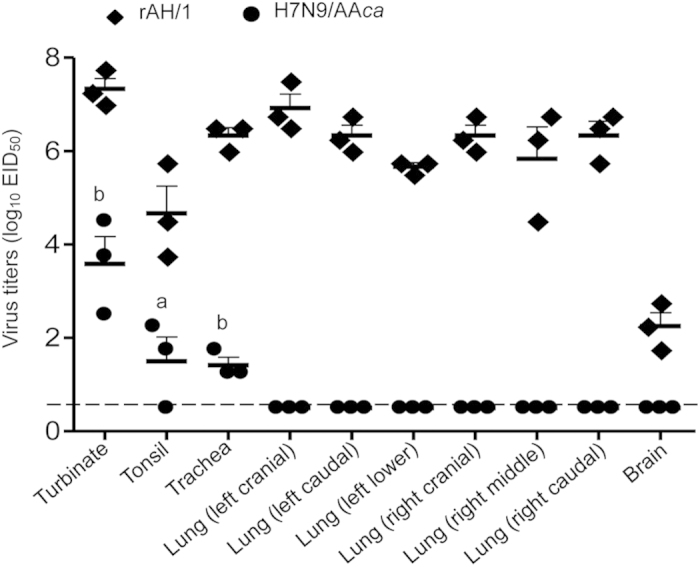
Attenuation of H7N9/AAca in ferrets. Groups of three ferrets were intranasally inoculated with 10^6^ EID_50_ of H7N9/AA*ca* or rAH/1. The animals were killed on day 4 p.i. and their nasal turbinates, tonsils, tracheas, lungs, and brains were collected for virus titration in eggs. The dashed line indicates the limit of detection. a, *P* < 0.05 and b, *P* < 0.01 compared with the corresponding value for rAH/1-inoculated animals.

The rAH/1 virus replicated efficiently in the nasal turbinates, tonsils, tracheae, lungs, and brains of ferrets, similarly to previous studies with the wild-type AH/1 virus^12^. The H7N9/AA*ca* virus, however, was detected in the nasal turbinates, tonsils, and tracheae of ferrets at significantly lower titers than those of the rAH/1 virus in ferrets, and was not detected in the lungs or brains of any ferrets (Fig. 2). These results indicate that the reassortant virus H7N9/AA*ca* was significantly attenuated in mammals relative to the wild-type AH/1virus.

### Immunogenicity and protective efficacy of the H7N9/AA*ca* vaccine in mice

The vaccine was introduced via the intranasal route into the animals tested in this study. Three weeks after inoculation of mice with a single dose of H7N9/AA*ca*, the mean HI and NT antibody titers against rAH/1 were 64 and 80, respectively. After the second vaccination, the HI and NT antibody titers increased sharply to 403 and 806, respectively (Table 1).

**Figure 3:**
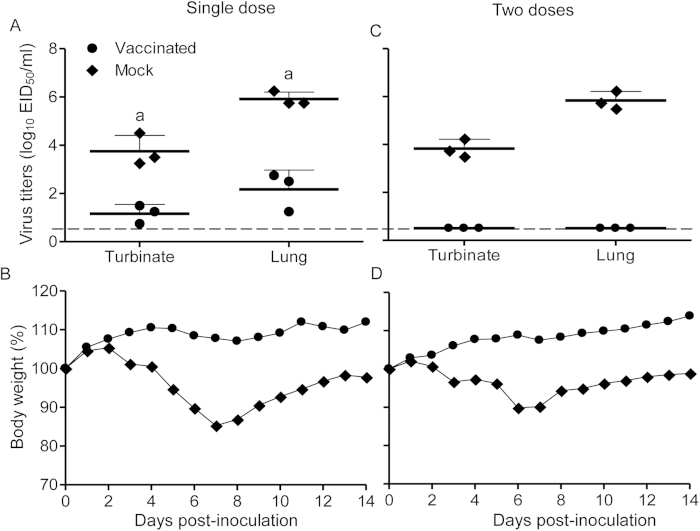
Protective efficacy of H7N9/AAca in mice. Groups of eight mice were challenged with 10^5^ EID_50_ of rAH/1 three weeks after being inoculated with one dose (**A** and **B**) or two doses (**C** and **D**) of H7N9/AA*ca*. Three mice were killed on day 3 post-challenge and their organs were collected for virus titration (**A** and **C**); the remaining five mice were observed for weight changes for 14 days (**B** and **D**). a, *P* < 0.05 compared with the corresponding value for PBS (mock)-inoculated animals.

Three weeks after one or two doses of vaccine, we challenged the mice intranasally with 10^5^ EID_50_ of rAH/1 virus. Three mice were killed on day 3 post-challenge (p.c.) and their organs were collected for virus titration; the remaining five mice in each group were observed for 2 weeks. As shown in Fig. 3, high titers of virus were detected in the nasal turbinates and lungs of unvaccinated mice, which also experienced up to 15% body weight loss (Fig. 3A–D). Virus was detected in the nasal turbinates and lungs of mice that received a single dose of vaccine; however, the titers were significantly lower than those of the mice in the control group (Fig. 3A). By contrast, mice that received two doses of vaccine were completely protected from rAH/1 virus challenge. Virus was not detected in any of the organs tested, and the mice remained healthy over the 2-week observation period (no weight loss) (Fig. 3C,D).

### Immunogenicity and protective efficacy of the H7N9/AA*ca* vaccine in ferrets

Three weeks after a single inoculation of ferrets with H7N9/AA*ca*, the mean HI and NT antibody titers against rAH/1 were 101 and 80, respectively. After the second vaccination, the mean HI and NT antibody titers increased sharply to 320 and 254, respectively (Table 1).

**Figure 4:**
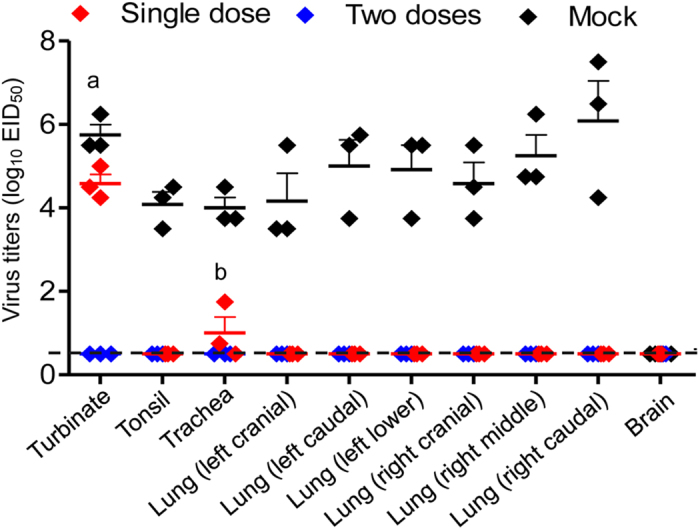
Protective efficacy of H7N9/AAca in ferrets. Ferrets were challenged with 10^5^ EID_50_ of rAH/1 three weeks after being inoculated with different doses of H7N9/AA*ca* or PBS. Organs were collected on day 4 post-challenge for titration in eggs. The dashed lines indicate the lower limit of detection. a, *P* < 0.05; b, *P* < 0.01 compared with the corresponding value for PBS (mock)-inoculated animals.

Three weeks after one or two doses of vaccine, we challenged the ferrets intranasally with 10^5^ EID_50_ of rAH/1 virus. The ferrets were killed on day 4 p.c. and their organs were collected for virus titration. As shown in Fig. 4, high titers of virus were detected in all tested organs of the unvaccinated ferrets. Virus was detected in the nasal turbinates and tracheae of ferrets that received a single dose of vaccine; however, the titers were significantly lower than those of the ferrets in the control group (Fig. 4). By contrast, ferrets that received two doses of vaccine were completely protected from rAH/1 virus challenge, and virus was undetectable in any of the organs tested (Fig. 4).

### Immunogenicity and protective efficacy of the H7N9/AA*ca* vaccine in guinea pigs

We also evaluated the protective efficacy of the H7N9/AA*ca* vaccine in the guinea pig model. As shown in Table 1, after a single dose of vaccine, the mean HI and NT antibody titers reached 64 and 80, respectively, and rose to 507 and 254, respectively, after the second dose of vaccine (Table 1).

**Figure 5:**
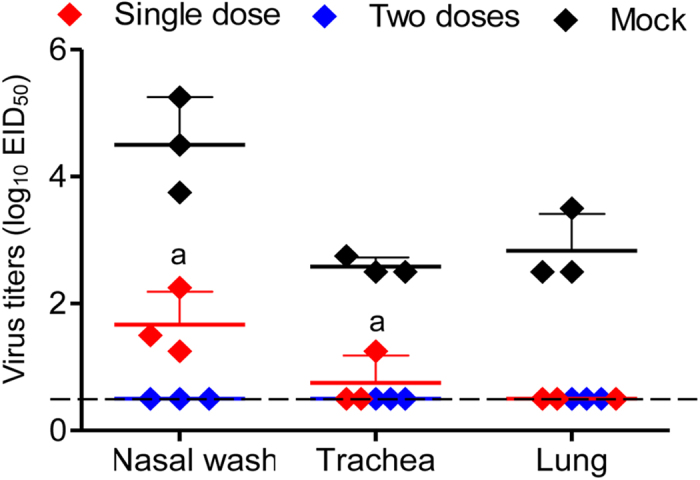
Protective efficacy of H7N9/AAca in guinea pigs. Guinea pigs were challenged with 10^5^ EID_50_ of rAH/1 three weeks after being inoculated with different doses of H7N9/AA*ca* or PBS. Organs were collected on day 3 post-challenge for titration in eggs. The dashed lines indicate the lower limit of detection. a, *P* < 0.05 compared with the corresponding value for PBS (mock)-inoculated animals.

Three days after challenge with 10^5^ EID_50_ of the rAH/1 virus, the animals were killed and their nasal washes, tracheae, and lungs were harvested for virus titration. Virus was detected in all tested samples collected from the control guinea pigs, and in the nasal washes and tracheae of the guinea pigs that received a single dose of vaccine at titers significantly lower than those in the control animals. Virus was undetectable in the guinea pigs that were vaccinated twice (Fig. 5).

### One dose of the H7N9/AA*ca* vaccine prevents the transmission of rAH/1

From the above results, we learned that the H7N9/AA*ca* vaccine could induce sound humoral immune responses in mice, ferrets, and guinea pigs, and that two doses of the vaccine could provide complete protection against H7N9 virus challenge in all three animal models tested. However, one 10^6^ EID_50_ dose of the H7N9/AA*ca* vaccine did not provide complete protection against H7N9 virus challenge in any of the animals, prompting us to investigate whether one dose of the vaccine could prevent infection by a natural route of transmission of H7N9 virus. We previously reported that the AH/1 virus transmits efficiently in ferrets^12^, and our preliminary data indicated that this virus also transmits efficiently in guinea pigs. Therefore, we used guinea pigs, a more economical model, to investigate whether the live attenuated H7N9 vaccinated animals could be protected from infection when they were exposed to or in contact with H7N9 virus-infected animals.

**Figure 6:**
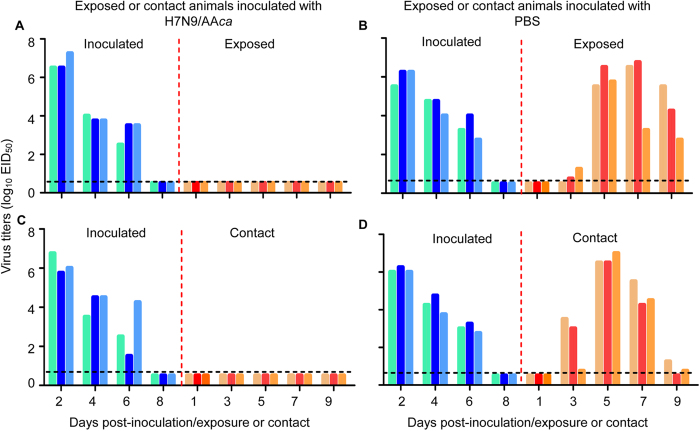
Respiratory droplet transmission of rAH/1 in guinea pigs. Groups of six guinea pigs were inoculated with 10^6^ EID_50_ of H7N9/AAca or with PBS. Three weeks later, three animals from each group were put in neighboring cages (**A** and **B**) (to monitor respiratory droplet transmission) or the same cages (**C** and **D**) (to monitor direct contact transmission) that hosted three guinea pigs that were inoculated with 10^6^ EID_50_ of rAH/1 virus. Each color bar represents a value from an individual animal. The dashed black lines indicate the lower limit of detection.

As shown in Fig. 6, virus was recovered on days 2, 4, and 6 post-inoculation (p. i.) from all guinea pigs that were inoculated with the rAH/1 virus, and from the exposed or contact guinea pigs that were previously inoculated with PBS on days 3, 5, 7, and 9 post-contact or post-exposure (Fig. 6B,D); however, virus was not detected from any of the exposed or contact animals that were previously vaccinated with H7N9/AA*ca* (Fig. 6A,C). These results indicate that a single dose of the H7N9/AA*ca* vaccine successfully protect the animals from infection by a natural route of H7N9 virus transmission.

## Discussion

We generated a reassortant H7N9 cold-adapted virus, H7N9/AA*ca*, by using reverse genetics and evaluated its immunogenicity and efficacy as a live attenuated vaccine. The virus was attenuated in mice and ferrets. The vaccine induced strong HI and NT antibody responses to H7N9 influenza virus in mice, guinea pigs, and ferrets, and after two immunizations, the vaccine completely protected these animals from challenge with H7N9 virus. We further demonstrated that a single immunization with the H7N9/AA*ca* vaccine could completely protect the animals from infection when they were exposed to or in contact with the H7N9 virus-infected animals. Our results suggest that the H7N9/AA*ca* virus merits testing in humans as a candidate live attenuated pandemic vaccine for use against H7N9 influenza virus.

The six internal genes of the AA*ca* virus have been widely used as the backbone for live attenuated vaccine development^22^,^23^,^24^,^25^,^26^,^27^,^28^,^29^; however, the two surface glycoproteins of the vaccine seed virus greatly affect the replication and immunogenicity of the vaccine seed virus. Fan *et al.* reported that live attenuated H5N1 vaccine bearing the HA and NA genes from a clade 7 H5N1 virus could not induce any detectable HI and NT antibody response even after two doses of the vaccine were inoculated into mice, whereas a vaccine bearing the HA and NA genes from a clade 2.3 virus was highly immunogenic in both mice and monkeys^26^. Matsuoka *et al.* tested cold-adapted H5N1, H7N3, H6N1, and H9N2 live vaccines in African green monkeys and found that the replication and immunogenicity of these vaccines differed among the different subtypes^34^. Our H7N9 cold-adapted reassortant H7N9/AA*ca* virus replicates well in eggs, reaching a titer of 10^8.3^ EID_50_/ml, and is immunogenic in mice, guinea pigs, and ferrets. However, Chen *et al.* reported that a cold-adapted H7N9 vaccine containing the HA and NA genes from wild-type AH/1 replicated poorly in eggs^30^.

Previous studies have indicated that a single dose of cold-adapted vaccine could completely prevent homologous H5N1, H9N2, or H1N1 virus challenge in mice or ferrets^22^,^26^,^29^. Although Chen *et al.* reported that a single dose of an H7N9 cold-adapted vaccine, with two amino acid changes (N133D and G198E) in the HA gene of the seed virus, could prevent the replication of H7N9 challenge virus in ferrets^30^. In our study, one dose of the H7N9/AA*ca* vaccine was not able to completely prevent the H7N9 challenge virus from replicating in any of the three animal models we tested. The difference in protective efficacy between our study and the study by Chen *et al.*^30^ may be attributable to differences in the amount of virus in the vaccine inoculum and/or in the animals used.

Rapid immune responses induced by vaccination are extremely important to control the spread of influenza during a pandemic. A single dose of the inactivated vaccine could not prevent the animals from being infected when they came in contact with the H7N9 virus-infected animals^19^; however, a single dose of the H7N9/AA*ca* vaccine did completely protect animals from such infection. Although the mechanism underlying the difference in protective efficacy between these two vaccines remains to be investigated, the ability to prevent infection by a natural route of transmission with a single dose is a bona fide advantage of the live attenuated H7N9 vaccine. The risk of reassortment of the vaccine virus with a circulating influenza virus, resulting in a novel subtype of influenza that could spread in the human population, is a noteworthy concern associated with the use of a live attenuated influenza vaccine bearing surface genes derived from a novel influenza virus subtype. Nevertheless, given the continued spread of H7N9 viruses in poultry, which poses an increasing threat to human health, and the efficacy of our vaccine in animal models, we believe that this H7N9 cold-adapted vaccine has potential as an effective H7N9 virus countermeasure and should be evaluated further in humans.

## Methods

### Facility and Ethics statements

All experiments were conducted in a biosecurity level 3+ laboratory approved by the Chinese Ministry of Agriculture. Animal studies were approved by the Review Board of Harbin Veterinary Research Institute, Chinese Academy of Agricultural Sciences. The study was carried out in strict accordance with the recommendation in the Guide for the Care and Use of Laboratory Animals. Protocols for the animal studies were approved by the Committee on the Ethics of Animal Experiments of the Harbin Veterinary Research Institute, Chinese Academy of Agricultural Sciences.

### Viruses

A/Anhui/1/2013 (H7N9) (AH/1) is one of the index H7N9 viruses isolated from humans^4^ and has been shown to transmit efficiently between ferrets by respiratory droplet^12^,^14^. Sanger sequencing revealed the presence of a mixture of viruses in the Anhui/1 stock^14^. Therefore, to avoid any biological effects due to mutations in the genome, we rescued the AH/1 virus from cloned genes by using reverse genetics^31^,^32^,^33^, and used the rescued virus (rAH/1) for our studies. The recombinant virus H7N9/AA*ca*, which bears HA and NA genes from AH/1 and its six internal genes from AA*ca* was also generated by reverse genetics^31^,^32^,^33^. The HA and NA genes were cloned from the AH/1 virus, whereas the PB2, PB1, PA, NP, M and NS genes of the AA*ca* virus were synthesized as previously reported^26^. Virus stocks were propagated in specific pathogen-free chicken eggs.

### Mouse studies

Six-week-old female specific-pathogen-free BALB/c mice were used in this study. First, the wild-type rAH/1 and reassortant H7N9/AA*ca* viruses were tested for their replicative capacity. Groups of eight mice were anesthetized with CO_2_ before being inoculated intranasally with 10^6^ 50% egg infectious dose (EID_50_) of the rAH/1 virus, the H7N9/AA*ca* virus, or PBS. Three mice from each group were killed on day 3 post-inoculation (p.i.), and their organs were harvested, homogenized, and titrated in 10-day-old embryonated eggs. Titers were calculated by using the Reed-Muench method and are expressed as log_10_ EID_50_/ml. The remaining five mice were observed daily for weight changes for 14 days.

For immunogenicity and vaccine studies, groups of eight mice were anesthetized with CO_2_ and intranasally inoculated with10^6^ EID_50_ of the H7N9/AA*ca* in 50 μl once or twice (three weeks apart) or with PBS as a control. Sera were collected 3 weeks after each vaccination from three animals in each group for hemagglutination inhibition (HI) assays and neutralization (NT) antibody detection using the homologous H7N9 virus rAH/1 as the antigen. Three weeks after vaccination, the mice were challenged with 10^5^ EID_50_ of rAH/1 intranasally; three mice from each group were killed on day 3 post-challenge (p.c.), and their organs were collected for virus titration. The remaining five mice were observed for 14 days for body weight changes and death.

### Ferret studies

Four-month-old female (Wuxi Cay Ferret Farm, Jiangsu, China) ferrets that were sero-negative were used in this study. To evaluate the replication ability of the rAH/1 and H7N9/AA*ca* viruses in this animal model, groups of three ferrets were inoculated with 10^6^ EID_50_ of rAH/1 or H7N9/AA*ca*. Each ferret was inoculated with 10^6^ EID_50_ of test virus in a volume of 500 μl (250 μl per nostril). Four days after inoculation, ferrets were euthanized and organs, including nasal turbinates, tonsils, different parts of the respiratory system, and brains, were harvested for virus titration in eggs. Titers were calculated by using the Reed-Muench method and are expressed as log_10_ EID_50_/g of tissue.

To evaluate the protective efficacy of H7N9/AA*ca*, groups of three 4-month-old female ferrets were intranasally inoculated once or twice with 10^6^ EID_50_ of H7N9/AA*ca* in 500 μl (250 μl per nostril) (three weeks apart) or with PBS as a control. Sera were collected at three weeks after each vaccination for HI and NT antibody detection. Three weeks after the first or second immunization, the animals were challenged by intranasal inoculation of 10^5^ EID_50_ of rAH/1 virus in a 500-μl volume. Four days later, the animals were euthanized, and organs, including turbinates, tonsils, and different parts of the respiratory system, were collected for virus titration. Titers were calculated by using the Reed-Muench method and are expressed as log_10_ EID_50_/g of tissue.

### Guinea pig studies

To evaluate the protective efficacy of the vaccine in the guinea pig model, groups of three female guinea pigs (VITAL RIVER) weighing 250–280 g were intranasally immunized with one or two doses of 10^6^ EID_50_ of H7N9/AA*ca* in 300 μl (150 μl per nostril) (three weeks apart) or with PBS as a control. Three weeks after vaccination, sera were collected for HI and NT antibody detection and the animals were challenged with 10^5^ EID_50_ of rAH/1. The guinea pigs were euthanized on day 3 p.c., their nasal washes, tracheae, and lungs were collected for virus titration in eggs.

To evaluate whether a single dose of the vaccine could prevent H7N9 virus infection by a natural route of transmission, groups of six guinea pigs were inoculated with 10^6^ EID_50_ of H7N9/AA*ca* in a volume of 300 μl (150 μl per nostril) or with PBS as a control. Three weeks later, three animals from each group were put into the same cage or a neighboring cage that hosted three guinea pigs that had been inoculated with 10^6^ EID_50_ of rAH/1 24 h before. Nasal washes were collected from all animals at 2-day intervals, beginning on day 2 p.i. (i.e., 1 day post-contact or -exposure), for 14 days and titrated in eggs. The ambient conditions for these studies were set at 20–22 ^o^C and 30%–40% relative humidity. The airflow in the isolator was horizontal with a speed of 0.1 m/s. For the respiratory droplet transmission study, the airflow direction was from the inoculated animals to the exposed animals.

### Antibody detection

Sera were treated with Vibrio cholera (Denka-Seiken, www.denka-seiken.co.jp) receptor-destroying enzyme before being tested for the presence of HI antibody with 0.5% (V/V) chicken erythrocytes. The neutralization (NT) antibody titers were determined in eggs with heat-inactivated sera collected from mice, guinea pigs, and ferrets. The cutoff value used for the HI and NT antibody assays was 10.

### Statistical analysis

Virus titers were compared by use of the Student’s t-test. Differences were considered significant when the *P* value was less than 0.05.

## Additional Information

**How to cite this article**: Kong, H. *et al.* A live attenuated vaccine prevents replication and transmission of H7N9 virus in mammals. *Sci. Rep.* **5**, 11233; doi: 10.1038/srep11233 (2015).

## Figures and Tables

**Table 1:** **Antibody responses induced by the H7N9/AA** ***ca*** **reassortant virus in mice, guinea pigs, and ferrets** a

**Animal**	**Mean antibody titers (range)**					
	**Pre-test**		**Dose 1**		**Dose 2**	
	**HI**	**NT**	**HI**	**NT**	**HI**	**NT**
Mouse	<10	<10	64 (40–80)	80 (80–80)	403 (320–640)	806 (640–1280)
Ferret	<10	<10	101 (80–160)	80 (40–160)	320 (320–320)	254 (160–320)
Guinea pig	<10	<10	64 (40–80)	80 (80–80)	507 (320–640)	254 (160–320)

^a^The indicated animals were inoculated with one or two doses, with a 3-week interval, of 10^6^ EID_50_ of the H7N9/AA*ca* virus. Three weeks after dose 1 or dose 2, sera were collected to determine HI and NT antibody titers by using the homologous wild-type H7N9 virus.
